# Mediterranean Diet Score: Associations with Metabolic Products of the Intestinal Microbiome, Carotid Plaque Burden, and Renal Function

**DOI:** 10.3390/nu10060779

**Published:** 2018-06-16

**Authors:** Michael Pignanelli, Caroline Just, Chrysi Bogiatzi, Vincent Dinculescu, Gregory B. Gloor, Emma Allen-Vercoe, Gregor Reid, Bradley L. Urquhart, Kelsey N. Ruetz, Thomas J. Velenosi, J. David Spence

**Affiliations:** 1 Schulich School of Medicine and Dentistry M.D. Candidate (CIHR Summer Research Training Program), London, ON N6A 3K7, Canada; mpignanelli2018@meds.uwo.ca; 2 Division of Neurology, Schulich School of Medicine and Dentistry, Western University, London, ON N6A 3K7, Canada; cjust2@uwo.ca; 3 Stroke Prevention & Atherosclerosis Research Centre, Robarts Research Institute, Western University, 1400 Western Road, London, ON N6G 2V4, Canada; chrysi.bogiatzi@medportal.ca; 4 Current address: Department of Neurology, McMaster University, Hamilton, ON L8S 4K1, Canada; 5 Department of Radiology, University of Calgary, Calgary, AB T2N 4N1, Canada; Vince.Dinculescu@uottawa.ca; 6 Department of Biochemistry, Schulich School of Medicine and Dentistry, Western University, London, ON N6A 5C1, Canada; ggloor@uwo.ca; 7 Department of Molecular and Cellular Biology, University of Guelph, Guelph, ON N1G 2W1, Canada; eav@uoguelph.ca; 8 Departments of Urology and Microbiology, Schulich School of Medicine and Dentistry, Western University, London, ON N6A 5C1, Canada; gregor@uwo.ca; 9 Departments of Physiology & Pharmacology, Schulich School of Medicine and Dentistry Western University, London, ON N6A 3K7, Canada; burquha2@uwo.ca (B.L.U.); Kruetz2@uwo.ca (K.N.R.); Tvelenosi@gmail.com (T.J.V.); 10 Divisions of Neurology and Clinical Pharmacology, Western University, London, ON N6A 5A5, Canada

**Keywords:** intestinal microbiome, metabolites, Mediterranean diet, TMAO, carotid plaque, renal function

## Abstract

Metabolic products of the intestinal microbiome such as trimethylamine N-oxide (TMAO) that accumulate in renal failure (gut-derived uremic toxins, GDUTs) affect atherosclerosis and increase cardiovascular risk. We hypothesized that patients on a Mediterranean diet and those consuming lower amounts of dietary precursors would have lower levels of GDUTs. Patients attending vascular prevention clinics completed a Harvard Food Frequency Questionnaire (FFQ) and had plasma levels of TMAO, p-cresylsulfate, hippuric acid, indoxyl sulfate, p-cresyl glucuronide, phenyl acetyl glutamine, and phenyl sulfate measured by ultra-performance liquid chromatography coupled to quadrupole time-of-flight mass spectrometry. Carotid plaque burden was measured by ultrasound; CKD-Epi equations were used to estimate the glomerular filtration rate. In total, 276 patients completed the study. Even moderate renal function significantly increased plasma GDUTs, which were significantly associated with higher carotid plaque burden. There was no significant difference in plasma levels of any GDUT associated with a Mediterranean diet score or with intake of dietary precursors. In omnivorous patients with vascular disease, the intake of dietary precursors of intestinal metabolites or adherence to a Mediterranean diet did not change plasma GDUT. Approaches other than diet, such as probiotics and repopulation of the intestinal microbiome, may be required to mitigate the adverse effects of GDUTs.

## 1. Introduction

The role of the intestinal microbiome in human health [1], and interactions between the diet, the intestinal microbiome, and vascular disease [2], as well as chronic kidney disease (CKD) [3] have become increasingly apparent in recent years. Trimethylamine is naturally abundant in fish [4]; it is also produced by intestinal bacteria from carnitine (largely from red meat) and phosphatidylcholine (largely from egg yolk). Trimethylamine is then oxidized in the liver to trimethylamine n-oxide (TMAO) [5,6]. In animal models, TMAO causes atherosclerosis [5]. In patients referred for coronary angiography, levels of TMAO after a test dose of two hard-boiled eggs significantly predicted cardiovascular risk: patients in the top quartile of TMAO levels had a 2.5-fold increase in the three-year risk of stroke, myocardial infarction, or vascular death [7].

One of the most intriguing aspects of this case is that the production of TMAO was affected by diet. When carnitine was given to meat-eaters, they produced TMAO; however, vegans given carnitine did not [6]. This suggests that modification of diet to reduce production of intestinal microbiome metabolic products may be a way to reduce cardiovascular risk. Another important aspect of this issue is that the plasma intestinal microbiome metabolites are renally excreted, so in renal failure, blood levels are high. High levels of TMAO accelerate decline of renal function and are associated with increased cardiovascular risk [8]. In patients with CKD, plasma levels of indoxyl sulfate (IS) and p-cresyl sulfate (PCS) are 54 and 17 times higher, respectively, than in healthy individuals [9]. Although protein binding is low, concentrations of phenylacetylglutamine concentrations are >100-fold higher in patients with end stage kidney disease [10]. 

Metabolic products of the intestinal microbiome, such as p-cresyl sulfate from tyrosine and phenylalanine, have been shown to be markers of abnormal microbiota present in the human intestine. P-cresyl sulfate (PCS) accelerates atherosclerosis and renal impairment by increasing oxidative stress and is associated with cardiovascular risk in relation to renal impairment. Other metabolic products of the intestinal microbiome, including phenylacetylglutamine, indole 3-acetic acid, p-cresyl sulfate, indoxyl sulfate, and p-cresyl glucuronide also accelerate cardiovascular disease in renal failure [11,12,13,14] Indoxyl sulfate and indole 3-acetic acid, derived from microbial metabolism of tryptophan, promote oxidative stress and endothelial dysfunction [15] and are associated with vascular stiffness, aortic calcification, and cardiovascular mortality in hemodialysis patients [11,16].

The Mediterranean diet is thought to be beneficial because it involves a high intake of fiber, vitamins, and antioxidants [17]. It appears that the eating pattern is what matters, as supplementation with individual vitamins and antioxidants does not appear to have as great an effect on cardiovascular risk as the Mediterranean diet.

In this study we assessed the intake of specific nutrient precursors and also the effect of Mediterranean diet scores on the plasma levels of seven intestinal microbiome metabolic products. As a “mainly vegetarian diet” [18], the Cretan Mediterranean diet would be expected to lower levels of all the intestinal metabolites we measured, since the components are derived from amino acids, carnitine, or choline. We also studied the association of renal function with plasma levels of the metabolites, and carotid plaque burden in relation to plasma levels of the metabolites and the Mediterranean diet score. We hypothesized that a low intake of specific nutrients and high Mediterranean diet scores would be associated with lower plasma levels of the metabolites and less of a carotid plaque burden.

## 2. Methods and Materials

### 2.1. Study Population

Patients enrolled in the study were identified from the clinical database of the Stroke Prevention and Atherosclerosis Research Centre, Robarts Research Institute, Western University, London, Ontario, Canada. Patients in the database were from one of several clinics at the University Hospital, London, Ontario, Canada: the Stroke Prevention Clinic, the Urgent Transient Ischemic Attack (TIA) Clinic, and the Premature Atherosclerosis Clinic. All patients were advised at the time of their first referral and at follow-up visits to follow a diet similar to the Mediterranean diet, counseled on how to achieve this, and given a recipe booklet with many websites listed where they could obtain additional recipes. They were advised to consume only whole grains, seldom consume red meat (e.g., once a month), use olive oil and replace butter with non-hydrogenated canola/olive oil margarine, consume a high intake of vegetables, fruits and legumes (e.g., lentils, beans, nuts, peas, chickpeas), and keep intake of animal flesh (mainly fish and chicken) to ~2 ounces daily or a serving the size of the palm of the hand every other day, avoiding egg yolks and limiting red meat. The recipe booklet is available for download from [19]. No patients received monetary compensation for participation in the study. They were recruited for a study of the intestinal microbiome in extremes of atherosclerosis [20] from a sample of 3056 patients with all data required to calculate residual scores in a regression model, in order to identify outliers with atherosclerosis not explained by traditional risk factors [20]. In that study 316 patients were recruited; in this paper we report the results of nutrient analyses for the 276 patients who completed the dietary assessment, as described below. Patients were enrolled between 2014 and 2016. The size of the study population was determined by funding available.

### 2.2. Dietary Assessment

Food intake over the past year was assessed using the blue 131-item self-reported and semi-quantitative Harvard Food Frequency Questionnaire (FFQ DA 80), which has been extensively validated [21,22]. As described previously, respondents were asked to select one of nine levels for each standardized food item, based on average frequency of consumption in the past year [23]. The nine options ranged from “never, or less than once per month” to “six or more times per day”. For evaluation of each nutrient, FFQ scores were converted into estimated physical quantity consumed. Using an approach outlined previously, the estimated number of servings per day was multiplied by standard serving size for each food [24]. 

Unless otherwise indicated, average nutrient intake was calculated by multiplying nutrient values (by mass) from the Harvard University Food Composition Database and the USDA National Nutrient Database for Standard Reference [23,25,26]. Results from each source of a particular nutrient were added to represent estimated total intake for specific nutrients. Total energy (in kcal) was determined using an analogous approach. Except for the estimation of free l-carnitine from diet, nutrient and energy coding was performed at the Channing School of Public Health.

### 2.3. Diet Patterns and Nutrient Scores

Adherence to the prescribed Mediterranean diet (associated with lower risk of incident CVD and stroke) was assessed by generating adherence to Mediterranean (aMED) diet scores from the FFQ [26,27]. The FFQ was administered at the time of enrolment in the study. Briefly, the aMED score is a composite score composed of an individual’s sum of specific items in a food group compared to the median of the summed frequency for the same food group. Food groups used to generate the aMED score included vegetables (excluding potatoes), fruits, nuts and seeds, red and processed meats, whole grains and plain cereals, fish, monounsaturated to saturated fat ratio, and ethanol intake. A score closer to eight represented greater adherence to the Mediterranean diet, whereas a score of 0 represented the lowest possible score. Patient intake of items from food groups associated with positive health outcomes received a score of 1 if their intake was above the median for that food group. Only red and processed meat intake received a 0 if patient intake surpassed the median intake of the sample. We also assigned 1 point for alcohol intake between 5–15 g/day, representing one 12 oz can of non-light beer, 5 oz of wine, or 1.5 oz liquor.

### 2.4. Total Protein, Amino Acids, Fiber and TMAO Precursors Including l-carnitine

Total protein and amino acids consumed per day were determined by summation of their density in each item on the FFQ as described above. Total indigestible fiber determination was accomplished using biochemical and/or enzymatic methods approved by the Association of Analytic Communities (AOAC) [28]. Total choline-containing nutrients included the sum of water-soluble choline-containing compounds (free choline, glycerophosphocholine, phosphocholine), lipid membrane-associated choline (phosphatidylcholine, sphingomyelin), and total betaine (without supplementation).

The following items from the FFQ were used to estimate total free l-carnitine: “chicken sandwich”, “chicken without skin”, “chicken liver”, “liver”, “bacon”, “extra-lean hamburger”, “hamburger”, “pork”, “beef or lamb as a main dish”, “tuna”, “shrimp”, “dark oily fish” [29]. Although not exhaustive for all sources of l-carnitine, these entries were selected because meat products are the most important sources of l-carnitine in human diets [30]. Concentrations of free l-carnitine in individual food items were estimated using values published by Demarquoy et al. [31], and online from the Linus Pauling Institute micronutrient database at Oregon State University [31,32,32]. Total trimethylamine precursor nutrients were calculated as the sum of choline-containing nutrients and l-carnitine intake per day.

### 2.5. Biochemical Methods

Plasma samples were obtained after an overnight fast at enrollment; after centrifugation they were stored at −80 °C until they were transported on dry ice to the biochemical laboratory. Metabolites produced by the intestinal microbiome were measured by ultra-performance liquid chromatography (UPLC) coupled to quadrupole time-of-flight mass spectrometry (QToF/MS), as previously described [20].

TMAO was determined by hydrophilic interaction liquid chromatography using methods similar to those previously published [33,34]. In brief, plasma proteins were precipitated with the addition of ice-cold acetonitrile (containing 10 µM betaine-d3 as internal standard) at a ratio of 4:1 acetonitrile:plasma. Addition of the acetonitrile containing betaine-d3 internal standard occurred prior to sample extraction. Following centrifugation, samples were concentrated 10-fold by evaporating the supernatant and reconstituting in acetonitrile. Reconstituted samples were transferred to vials and 10 µL were injected onto a Waters ACQUITY BEH Amide column (Waters Canada, Mississauga, ON, Canada) (1.7 µm, 2.1 × 100 mm). The mobile phases used for the analysis were 5 mM ammonium formate pH 3.5 (A) and acetonitrile (B), delivered in a gradient at a flow rate of 0.45 mL/min [34]. TMAO was measured by positive electrospray ionization and the Waters Xevo G2-S instrument for QToF/MS was operated in sensitivity mode. The 2M ± H ion (151.1446 *m*/*z*) was monitored for quantification of TMAO and the M ± H ion (121.1056 *m*/*z*) of internal standard betaine-d3 was monitored. Data were acquired in centroid mode with scans every 0.1 s. Accurate mass was confirmed using leucine-enkephalin (1 ng/µL) as a lockspray infused at a flow rate of 10 µL/min and scans acquired every 10 s.

**Figure 1:**
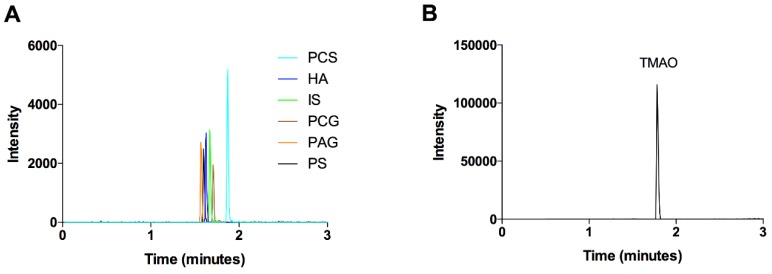
Representative chromatograms of metabolites of the intestinal microbiome. (**A**) Extracted ion chromatograms for p-cresyl sulfate (PCS), hippuric acid (HA), indoxyl sulfate (IS), p-cresyl glucuronide (PCG), phenylacetylglutamine (PAG), and phenyl sulfate (PS) were overlaid. These metabolites were measured using reverse phase liquid chromatography. (**B**) Extracted ion chromatogram of trimethylamine N-oxide (TMAO) which was measured by hydrophilic interaction liquid chromatography.

All other metabolites (p-cresyl sulfate, p-cresyl glucuronide, indoxyl sulfate, hippuric acid, phenylacetylglutamine, and phenyl sulfate) were measured using reversed phase chromatography as previously described [35]. Briefly, plasma proteins were precipitated by adding acetonitrile (containing 2.5 µM chlorpropamide as internal standard) in a ratio of 3:1 acetonitrile:plasma. Addition of the acetonitrile containing chlorpropamide internal standard occurred prior to sample extraction. Samples were centrifuged, and the supernatant was diluted 5-fold in water. Samples were transferred to vials and 1 μL was injected onto a Waters ACQUITY HSS T3 column (1.8 μm, 2.1 × 100 mm). The mobile phases used were water (A) and acetonitrile (B) both containing 0.1% formic acid, delivered at a flow rate of 0.45 mL/min. Column temperature was 45 °C. Metabolites were measured on a Waters Xevo G2-S apparatus for QToF/MS by negative electrospray ionization operating in resolution mode. The M−H ion for each metabolite was monitored: p-cresyl sulfate (187.0065 *m*/*z*), p-cresyl glucuronide (283.0822 *m*/*z*), indoxyl sulfate (212.0018 *m*/*z*), hippuric acid (178.0503 *m*/*z*), phenylacetylglutamine (263.1032 *m*/*z*), and phenyl sulfate (172.9908 *m*/*z*). Data were acquired in centroid mode with scans every 0.1 s. Accurate mass was confirmed using leucine-enkephalin (1 ng/µL) as a lockspray infused at a flow rate of 10 µL/min and scans acquired every 10 s. Inter and intra-day precision for all metabolites was less than 15%. Figure 1 shows a representative chromatogram.

Serum creatinine, total cholesterol, triglycerides, HDL cholesterol and LDL cholesterol were measured in the Biochemistry lab of the London Health Sciences Centre by routine methods. Renal function was estimated by the Chronic Kidney Disease Epidemiology Collaboration (CKD-Epi) equations.

### 2.6. Ethics

Participants gave written informed consent to an ethics protocol approved by the Western University Health Sciences Research Ethics board (approval number 12107E).

### 2.7. Statistical Methods

Metabolites produced by the intestinal microbiome were divided into quintiles, and nutrient intakes compared by quintile of metabolites with analysis of variance. Post hoc testing was performed using Tukey’s b test. Mediterranean diet scores were divided into quartiles or tertiles and compared by analysis of variance. These choices were made based on inspection of the data. Baseline characteristics of the study population were characterized as mean, SD for continuous variables and *n*, % for categorical variables. Statistical analyses were performed using SPSS (version 24, IBM Corporation, Armonk, NY, USA).

## 3. Results

The FFQ was completed by 276 study participants. Table 1 shows the patient characteristics; they were typically middle-aged/elderly high-risk vascular patients with reasonably well-controlled risk factors. The carotid plaque burden was very high (186.06 ± 221.84 mm^2^), which is in the top quartile of total plaque area in our clinic population, with a 19.5% five-year risk of stroke, myocardial infarction, or vascular death after adjustment for risk factors [36]. 

### 3.1. Components of the Mediterranean Diet Score

Table 2 shows the intake of the components of the Mediterranean diet score quintile of the aMed score. Except for alcohol, there were significant differences of the intake of all the other components. 

### 3.2. Intake of Nutrient Precursors of the Intestinal Metabolites

Supplemental Table S1 shows the intake of nutrient precursors of the intestinal metabolites by quintiles of metabolite levels; Table S2 shows nutrient intakes by quintile of TMAO levels. Despite very large differences in the plasma concentrations by quintiles of the metabolites, in general there were no significant differences in the intake of dietary precursors. An exception was for intake of vegetables and vegetable protein: lower intake was associated with lower plasma concentrations of phenylsulfate.

### 3.3. Renal Function and Plasma Levels of Intestinal Metabolites

Table 3 shows plasma levels of the intestinal metabolites by quartiles of eGFR; they were increased significantly with even modest renal impairment.

### 3.4. Carotid Plaque Burden by Quartiles of Plasma Levels of Intestinal Metabolites

**Figure 2:**
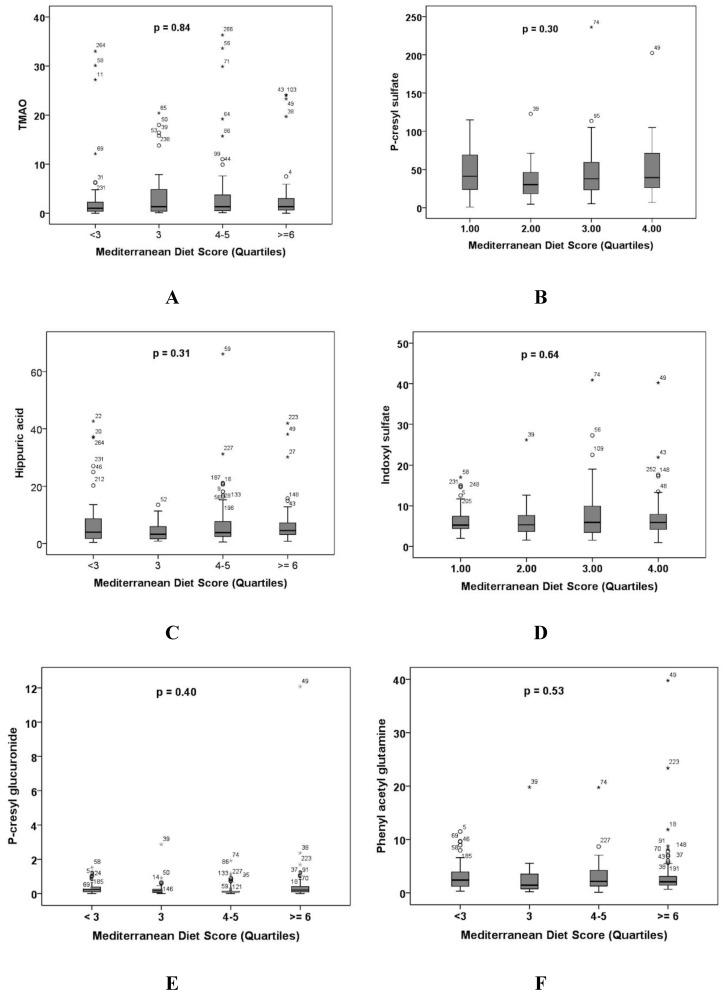

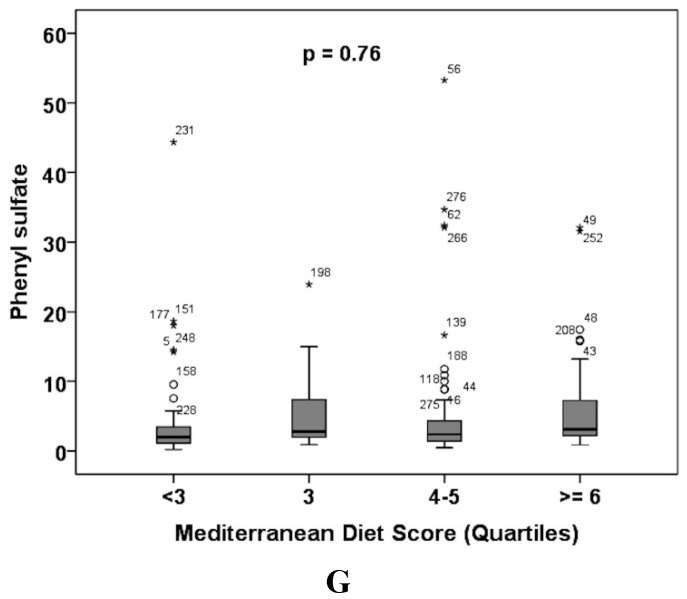
Carotid plaque burden is related to plasma levels of the intestinal metabolites. All the plasma levels are in µmol/L. TPA = total plaque area (mm^2^). (**A**) Trimethylamine N-oxide; (**B**) p-cresyl sulfate; (**C**) hippuric acid; (**D**) indoxyl sulfate; (**E**) p-cresyl glucuronide; (**F**) phenyl acetyl glutamine; and (**G**) phenyl sulfate. Differences were significant for trimethylamine N-oxide (*p* = 0.004), p-cresyl sulfate (*p* = 0.001), and phenyl acetyl glutamine *p* = 0.0001). ° refers to outliers; * refers to extreme outliers.

Figure 2 shows carotid plaque burden (total plaque area in mm^2^) by quartiles of plasma levels of the intestinal metabolites. 

**Figure 3:**
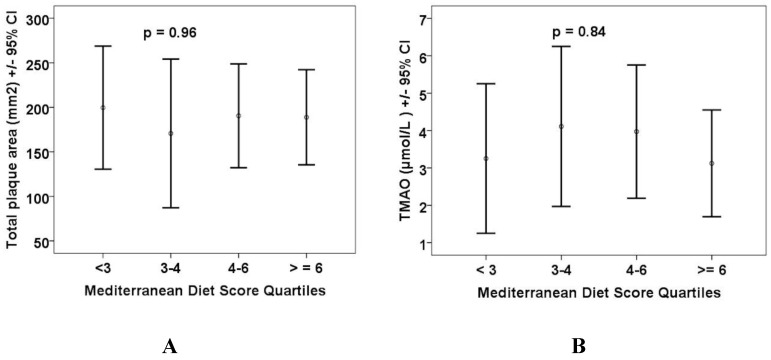

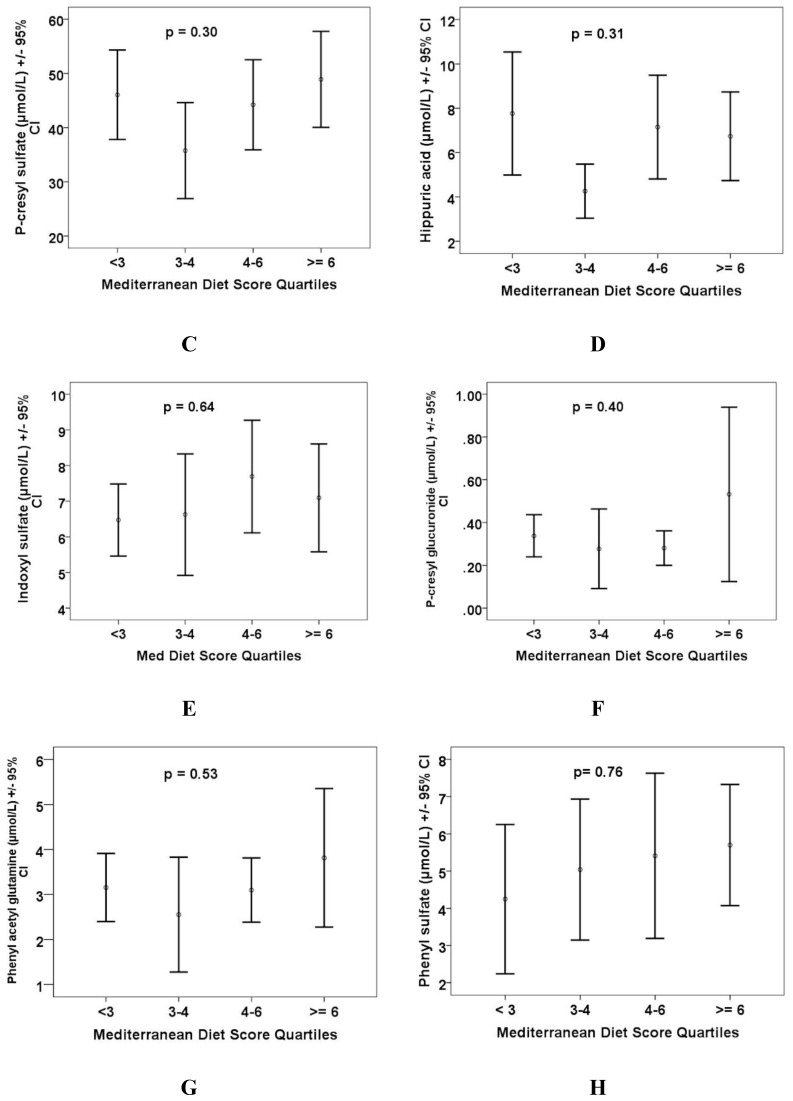
Carotid plaque burden and levels of intestinal metabolites by quartile of the Mediterranean diet score. Neither carotid plaque burden, nor the plasma levels of any of the metabolites, were significantly different by quartiles of the Mediterranean diet score. (**A**) Total plaque area (mm^2^); (**B**) trimethylamine N-oxide; (**C**) p-cresyl sulfate; (**D**) hippuric acid; (**E**) indoxyl sulfate; (**F**) p-cresyl glucuronide; (**G**) phenyl acetyl glutamine; and (**H**) phenyl sulfate.

Figure 3 shows carotid plaque burden and plasma levels of the intestinal metabolites by quartiles of the Mediterranean diet score.

In this small sample we did not find any differences in the intestinal microbiome related to atherosclerosis severity or diet (submitted to Data in Brief as a companion article to a report in Atherosclerosis) [20].

## 4. Discussion

We found that among omnivore vascular patients, neither the intake of specific nutrient precursors, nor the Mediterranean diet score predicted plasma levels of the intestinal metabolites. This suggests that it is the makeup of the intestinal microbiome that is the main determinant of the production of these metabolites. Even modestly impaired renal function resulted in significantly higher plasma levels of the metabolites, and carotid plaque burden was higher in patients with higher plasma levels of the intestinal metabolites. Renal impairment may also alter the intestinal flora [37]. In our study, renal function was better than in most earlier studies relating to the intestinal metabolites. In the study of Tang et al. [8], in which it was reported that high levels of TMAO accelerated decline of renal function and increased cardiovascular risk, the participants had an estimated glomerular filtration rate (eGFR) <60. In the study of Stubbs et al. [38], elevated levels of TMAO were seen only in participants with advanced kidney disease. In the present study, carotid plaque burden was not related significantly to the Mediterranean diet score.

Some studies have reported an effect of the Mediterranean diet on the intestinal microbiome [39,40]. De Filippis et al. studied 51 vegans, 51 vegetarians, and 51 omnivores, of which 30% had a high adherence to a Mediterranean diet. They found differences in plasma levels of fatty acids, and vegans and vegetarians had lower plasma levels of TMAO. Mitsou et al. [39] studied 120 Greek persons and found differences in some bacteria and in features of stool and bowel habits, but did not study intestinal metabolites. Perhaps our results were different because we studied patients with severe atherosclerosis, none of our study participants was vegan or strictly vegetarian, and few had high adherence to the Mediterranean diet.

An important limitation of our study was that the plasma levels of the metabolites were measured in the fasting state. In the study of Tang et al. in patients referred for coronary angiography, plasma levels of TMAO were measured after a test dose of two hard-boiled eggs [7] (containing ~500 mg of phosphatidylcholine); they reported that plasma TMAO increased for ~8 h after consuming egg yolk. Miller et al. [41] reported a steep dose–response relationship between egg consumption and plasma TMAO. A major limitation was that our study was cross-sectional; ideally patients would be crossed over in random sequence from usual diet to a Mediterranean diet and vice-versa. Another important limitation of our study is that there were no vegans in our patient population; it would be important to study the questions we addressed in this study in vegans. Also, we had no data on physical activity, which may be associated with the benefits of the Mediterranean diet. High-level adherence to a Mediterranean diet has been reported to have beneficial effects on the intestinal microbiome [40], however in our study only three participants achieved the highest Mediterranean diet score of 8; the median score in the top quintile of Mediterranean diet score was 6. Nevertheless, this level of adherence is probably better than that of the average North American. The American Heart Association reported in 2015 that 91.6% of Americans follow a poor diet, only 0.1% follow a healthy diet, and only 8.3% follow an intermediate diet [42]. This highlights the challenges of persuading vascular patients, many of whom have followed a poor diet all their life, to adhere to a healthy diet. A possible factor is the cost of fresh fruits and vegetables, and olive oil.

Levels of TMAO can be reduced by inhibitors of flavin monooxygenase 3, the enzyme responsible for oxidation of trimethylamine to TMAO [43,44], but this may not mitigate the effects of other intestinal metabolites. In patients with renal failure, broader approaches to reducing levels of intestinal metabolites could include more intensive dialysis, and renal transplantation. Probiotics and prebiotics may be feasible approaches to treatment of this problem, particularly if strains can impact directly or indirectly these compounds. The ability of strains to affect serum cholesterol is an example of such activity [45]. Fecal transplantation, or implantation of a selected panel of benign microbes (as used to treat *Clostridiodes difficile* [46,47]) present possible novel approaches to treatment of atherosclerosis.

## 5. Conclusions

Even moderately impaired renal function was associated with significantly higher plasma levels of the intestinal metabolites, and carotid plaque burden was significantly higher in patients with higher plasma levels. Carotid plaque burden was not related to Mediterranean diet scores. Neither the intake of specific nutrient precursors nor the Mediterranean diet score significantly predicted plasma levels of the intestinal metabolites among omnivorous vascular patients. Other approaches to reduce the impact of the intestinal microbiome on cardiovascular disease may be required. These might include probiotics, prebiotics, or transplantation of stool or panels of beneficial bacteria.

## Figures and Tables

**Table 1:** Characteristics of the study population. *n* = 276.

Continuous Variables	Mean	SD	Range
Age (years)	66.87	10.45	40–91
Body mass index (kg/M^2^)	28.49	6.08	17.1–68.5
Systolic blood pressure (mmHg)	143.27	21.29	103–221
Diastolic blood pressure (mmHg)	84.11	12.20	53–142
Total cholesterol (mmol/L)	4.75	1.14	2.03–8.12
Triglycerides	1.76	1.07	0.37–7.33
HDL-C (mmol/L)	1.33	0.41	0.44–3.43
LDL-C (mmol/L)	2.61	1.01	0.58–5.86
eGFR CKD-Epi	75.95	19.73	6.43–112.54
Total plaque area (mm^2^)	186.06	221.84	0–975
Categorical variables	*n*	%	
Male	164	59.4%	
Diabetic	53	19.2%	
Smoker			
Never	105	38.0%	
Quit	153	55.4%	
Still smoking	18	6.5%	

**Table 2:** Components of the Mediterranean diet score. All components are shown as grams per day except for the ratio of monounsaturated to saturated fats.

	Quintiles of the Mediterranean Diet Score					
Quintile Score	Q1 <2	Q2 2–4	Q3 4	Q4 5	Q5 6	*p* Value *
Alcohol (g)	9.45 ± 14.66	11.00 ± 15.66	13.88 ± 19.35	12.36 ± 21.18	9.77 ± 13.59	0.78
Monounsaturated to saturated fat ratio	0.95 ± 0.11	1.12 ± 0.29	1.19 ± 0.29	1.27 ± 0.50	1.43 ± 0.35	0.0001
Fish	2.21 ± 1.25	2.72 ± 1.73	3.31 ± 1.78	5.89 ± 8.05	9.92 ± 10.05	0.0001
Red/processed meat	18.93 ± 9.03	22.10 ± 11.71	19.28 ± 11.00	25.64 ± 13.82	38.72 ± 31.36	0.0001
Whole grains	5.43 ± 3.96	8.97 ± 6.34	10.18 ± 7.49	13.25 ± 7.91	23.52 ± 14.33	0.0001
Legumes	2.57 ± 2.13	6.01 ± 4.51	7.90 ± 6.11	9.82 ± 5.86	18.10 ± 10.84	0.0001
Fruit	8.57 ± 3.84	10.48 ± 7.14	13.41 ± 8.48	22.36± 17.64	32.95 ± 21.29	0.0001
Vegetables	20.79 ± 8.22	28.57 ± 11.06	36.69 ± 11.78	48.96 ± 24.26	76.87 ± 47.37	0.0001
Nuts	1.93 ± 1.38	2.70± 1.98	4.08 ± 3.19	4.08 ± 3.19	9.40 ± 6.45	0.0001

* ANOVA; comparison across groups Post hoc Tukey’s b tests are presented in the Supplementary Material.

**Table 3:** Plasma levels of the intestinal metabolites by tertiles of estimated glomerular filtration rate.

	Tertiles of eGFR			
Plasma Levels of Metabolites (µmol/L)	T1 <71	T2 71–86	T3 >87	*p* Value *
TMAO	5.10 ± 8.08	3.45 ± 5.93	2.30 ± 5.83	0.02
P-cresyl sulfate	63.19 ± 38.26	40.44 ± 23.80	29.37 ± 178.11	0.0001
Hippuric acid	8.97 ± 10.86	5.47 ± 6.20	6.13 ± 8.26	0.024
Indoxyl sulfate	9.93 ± 7.0	5.72 ± 2.73	4.26 ± 2.08	0.0001
P-cresyl glucuronide	0.61 ± 1.33	0.27 ± 0.27	0.20 ± 0.23	0.002
Phenyl acetyl glutamine	4.79 ± 5.16	2.47 ± 1.76	1.93 ± 2.54	0.0001
Phenyl sulfate	7.10 ± 9.24	4.04 ± 5.54	3.07 ± 3.10	0.0001

* ANOVA; comparison across groups; eGFR = estimated glomerular filtration rate, calculated by the CKD-Epi equations) Post hoc Tukey’s b tests are presented in the Supplementary Material. TMAO = trimethylamine N-oxide

## Supplementary Materials

The following are available online at http://www.mdpi.com/2072-6643/10/6/779/s1, Table S1: Intake of dietary precursors by quintiles of metabolite levels, Table S2: Nutrient intakes by quintile of TMAO levels.
