# Supplementary material for: Mediterranean Diet Score: Associations with Metabolic Products of the Intestinal Microbiome, Carotid Plaque Burden, and Renal Function
Source: Nutrients. 2018 Jun 16;10(6):779. doi: 10.3390/nu10060779 (PMC6024790; doi:10.3390/nu10060779)
Supplement: Supplementary file 1 [file nutrients-10-00779-s001.pdf]

nutrients

Mediterranean diet score: associations with metabolic products of the intestinal microbiome, carotid plaque burden and renal function
Supplementary Material

Michael Pignanelli BMSc., Caroline Just M.D.,Chrysi Bogiatzi M.D., Vincent Dinculescu M.D.,Gregory Gloor Ph.D., Emma Allen-Vercoe Ph.D., Gregor Reid Ph.D., Bradley L. Urquhart Ph.D., Kelsey N. Ruetz BMSc., Thomas J. Velenosi PhD, J. David Spence M.D.

Calculation of L-carnitine intake from Harvard FFQ
Michael Pignanelli BMSc.

Food intake over the past year was assessed using the 131 item self-reported and semi-quantitative Harvard Food Frequency Questionnaire (FFQ). As described previously, respondents were asked to select one of nine levels for each standardized food item, based on average frequency of consumption in the past year. The nine options ranged from "never, or less than once per month" to "six or more times per day". Using an approach outlined by Dr. Walter Willett[1], estimated number of servings per day was calculated by converting patient responses as follows: 1 = 0.08, 2 = 0.14, 3 = 0.43, 4 = 0.8, 5 = 1.00, 6 = 2.50, 7 = 4.50, 8 = 6.00, and 9 = 0.00. Any missing values were assigned a "0" value indicating no consumption of that food item. The number of servings per day was then multiplied by standard serving size of each food to get total amount per day. When serving sizes were not explicit, assumptions were made based on food labels: 1 slice of bacon = 1 oz., shrimp = 3 oz., tuna = 4 oz., tuna or dark meat fish = 4 oz. The amounts per day for each food item was then converted to number of 100g servings.

The following items from the FFQ were used to estimate total free L-carnitine: "chicken sandwich", "chicken without skin", "chicken liver", "liver", "bacon", "extra-lean hamburger", "hamburger", "pork", "beef or lamb as a main dish", "tuna", "shrimp", "dark fish". Although not exhaustive for all sources of L carnitine, these entries were selected because meat products are the most important sources of L carnitine in human diets.[2] Estimated free L-carnitine density (mg/100g portion) of each individual food item was taken from values published by Demarquoy et al.[3], and online from the Linus Pauling Institute micronutrient database at Oregon State University (available online at *http://lpi.oregonstate.edu/mic/dietary-factors/Lcarnitine*), as needed.[3, 4]

For items on the FFQ that were not found, it was assumed those items held values similar to related food items. For instance, "turkey meat with skin" and "lean hamburger" were given the same carnitine density as "chicken meat with skin" and "regular hamburger", respectively. When cut, cooking method of a particular meat, or, more than two meat items were included in the same food item category, the mean carnitine density value for all involved items was calculated for use. All items were then converted to mg/day and summed into a total carnitine

number per-day. Calculation and data management was accomplished using RStudio (Version 0.99.902 – © 2009-2016 RStudio, Inc.).

References

1. Willett W. *Nutritional epidemiology*. Oxford University Press, USA; 1998.
2. Lombard KA, Olson AL, Nelson SE, Rebouche CJ. Carnitine status of lactoovovegetarians and strict vegetarian adults and children. *The American journal of clinical nutrition*. 1989;50:301-306
3. Demarquoy J, Georges B, Rigault C, Royer M-C, Clairet A, Soty M, et al. Radioisotopic determination of l-carnitine content in foods commonly eaten in western countries. *Food Chemistry*. 2004;86:137-142
4. Higdon J, Drake VJ, Hagen TM. L-carnitine. *Micronutrient Information Center*. 2002;2016

Supplemental Table 1. Intake of dietary precursors by quintiles of metabolite levels(5, 34-38)

(Mean in top row, SD in italics below)

TMAO = trimethyamine n-oxide; Pre-TMA = Pre-trimethylamine; total TMA precursor nutrients; the sum of choline-containing nutrients and L-carnitine intake per day.

Metabolite	Dietary Precursor	Quintile of metabolite level 1st	2nd	3rd	4th	5th	P (ANOVA)
**TMAO (μmol/L)**		0.28	0.62	1.18	2.48	13.46	P <0.0001
		0.13	*0.11*	*0.20*	*0.68*	*10.40*	
	Meat g/day	26.52	26.08	26.50	28.34	25.55	0.98
		16.00	*19.94*	*21.86*	*24.91*	*21.87*	
	Animal protein g/day	56.31	51.23	50.93	44.48	44.03	0.08
		30.05	*23.89*	*20.79*	*17.89*	*22.79*	
	Free carnitine mg/day	31.36	26.46	27.38	24.65	22.48	0.20
		22.11	*15.11*	*18.12*	*14.23*	*18.13*	
	Eggs Per week	4.41	3.74	5.14	3.48	3.57	0.43
		4.23	*5.04*	*4.90*	*4.63*	*4.61*	
	Betaine + choline mg/day	460.58	463.68	463.21	449.26	436.50	0.98
		208.69	*189.26*	*152.29*	*178.63*	*327.47*	
	Pre-TMA mg/day	496.91	501.31	481.19	474.96	462.83	0.94
		224.92	*191.01*	*152.57*	*187.12*	*349.49*	
**P-cresyl sulfate (μmol/L)**		11.40	26.67	38.07	55.98	93.29	P < 0.0001
		5.00	*3.86*	*3.55*	*8.36*	*30.46*	
	Animal protein	53.58	51.03	51.28	48.71	42.22	
		26.97	*24.51*	*23.94*	*22.33*	*21.00*	

**Hippuric Acid (µmol/L)**		1.19	2.46	3.96	6.62	19.41	0.0001
		0.48	*0.44*	*0.47*	*1.20*	*12.52*	
	Glycine g/day	3.57	2.98	3.11	3.24	3.11	0.35
		1.48	*1.65*	*1.24*	*1.27*	*1.37*	
**Indoxyl sulfate (µmol/L)**		2.39	3.98	5.39	7.21	14.14	0.0001
		0.69	*0.48*	*0.43*	*0.62*	*6.70*	
	Tryptophan g/day	0.86	0.82	0.89	0.90	0.79	0.56
		0.38	*0.36*	*0.35*	*0.46*	*0.28*	
**P-cresyl glucuronide (µmol/L)**		0.02	0.11	0.20	0.34	1.12	0.0001
		0.02	*0.03*	*0.02*	*0.06*	*1.60*	
	Tyrosine g/day	3.02	2.89	2.69	2.57	2.47	0.19
		1.31	*1.25*	*1.23*	*1.05*	*1.07*	
**Phenyl acetyl glutamine (µmol/L)**		0.62	1.33	2.13	3.17	8.02	0.0001
		0.22	*0.21*	*0.23*	*0.49*	*5.72*	
	Phenylalanine g/day	3.52	3.35	3.60	3.70	3.19	*0.51*
		1.53	*1.31*	*1.57*	*1.93*	*1.06*	
	Glutamine g/day	3.52	3.35	3.60	3.70	3.19	0.63
		1.53	*1.31*	*1.57*	*1.93*	*1.06*	
**Phenyl sulfate (µmol/L)**		0.86	1.64	2.52	4.16	14.47	0.0001
		0.26	*0.24*	*0.28*	*0.85*	*9.92*	
	Methionine g/day	1.62	1.64	1.83	1.83	1.73	0.56
		0.72	*0.75*	*0.87*	*0.82*	*0.61*	
	Vegetables g/day	36.11	38.95	57.96	48.56	46.36	0.04
		24.23	*31.11*	*44.44*	*28.91*	*36.98*	
	Vegetable protein g/day	26.06	24.43	29.46	33.82	28.69	0.04
		13.65	*10.93*	*12.74*	*22.04*	*11.85*	
	Fruits servings/day	1.00	2.00	3.00	4.00	5.00	0.06
		13.62	*16.83*	*24.53*	*19.05*	*19.31*	
	g/day						
	Meat g/day	49.45	31.14	23.32	26.84	19.10	
		23.94	*25.31*	*17.89*	*21.92*	*9.08*	
	Tyrosine g/day	2.83	2.95	2.83	2.69	2.41	
		1.29	*1.33*	*1.23*	*1.13*	*0.99*	

Supplementary Table 2. Nutrient intakes by quintile of TMAO levels

Descriptives

		Mean	Std. Deviation
Alanine (g)	1.00	3.9107	1.92167
	2.00	3.7976	1.51022
	3.00	3.7507	1.31236
	4.00	3.4364	1.24996
	5.00	3.3089	1.76578
	Total	3.6382	1.58413
Arginine (g)	1.00	4.6220	2.26824
	2.00	4.5346	1.76324
	3.00	4.5016	1.70670
	4.00	4.0345	1.51720
	5.00	3.9320	2.08962
	Total	4.3217	1.90237
Aspartate (g)	1.00	7.7162	3.41766
	2.00	7.6739	3.03350
	3.00	7.5756	2.62149
	4.00	6.9733	2.61003
	5.00	6.6804	3.65178
	Total	7.3170	3.10982
Cysteine (g)	1.00	.9911	.47420
	2.00	.9763	.39472
	3.00	.9512	.31029
	4.00	.9164	.37612
	5.00	.8648	.46906
	Total	.9392	.41012
Glutamate (g)	1.00	16.0664	7.51391
	2.00	15.9671	6.57589
	3.00	15.2191	5.25256

	4.00	14.4200	6.15423
	5.00	13.6457	7.65947
	Total	15.0479	6.72031
Glycine (g)	1.00	3.4309	1.73802
	2.00	3.3695	1.35987
	3.00	3.2993	1.17542
	4.00	3.0274	1.13268
	5.00	2.8824	1.50288
	Total	3.1988	1.40936
Histidine (g)	1.00	2.3918	1.16500
	2.00	2.2956	.93018
	3.00	2.2337	.81269
	4.00	2.0436	.76849
	5.00	1.9750	1.04039
	Total	2.1865	.96317
Isoleucine (g)	1.00	3.7829	1.81180
	2.00	3.6685	1.48636
	3.00	3.5558	1.28087
	4.00	3.2788	1.26978
	5.00	3.1489	1.61973
	Total	3.4843	1.51909
Leucine (g)	1.00	6.5507	3.08309
	2.00	6.3485	2.56197
	3.00	6.1935	2.24325
	4.00	5.6648	2.21887
	5.00	5.4617	2.78676
	Total	6.0394	2.61885
Lysine (g)	1.00	5.9189	2.94185
	2.00	5.6239	2.31294
	3.00	5.5356	2.00737
	4.00	5.0226	1.86655
	5.00	4.8789	2.49196
	Total	5.3933	2.37633
Methionine (g)	1.00	1.9029	.93924
	2.00	1.8112	.74088
	3.00	1.7765	.63853
	4.00	1.6129	.60450

	5.00	1.5617	.77918
	Total	1.7321	.75702
Phenylalanine (g)	1.00	3.7122	1.70249
	2.00	3.6598	1.46165
	3.00	3.5498	1.27819
	4.00	3.2760	1.31208
	5.00	3.1378	1.61936
	Total	3.4639	1.49263
Proline (g)	1.00	5.4316	2.61331
	2.00	5.3763	2.35643
	3.00	5.0733	1.97905
	4.00	4.7395	2.16894
	5.00	4.4676	2.38379
	Total	5.0118	2.32336
Serine (g)	1.00	3.9520	1.80548
	2.00	3.8568	1.54594
	3.00	3.7621	1.35874
	4.00	3.4448	1.38634
	5.00	3.3226	1.75145
	Total	3.6648	1.59056
Threonine (g)	1.00	3.2184	1.51528
	2.00	3.1146	1.24907
	3.00	3.0691	1.07847
	4.00	2.8343	1.07223
	5.00	2.7080	1.45278
	Total	2.9867	1.29516
Tryptophan (g)	1.00	.9149	.42421
	2.00	.8941	.35802
	3.00	.8695	.29060
	4.00	.8205	.33720
	5.00	.7761	.40937
	Total	.8543	.36871
Tyrosine (g)	1.00	2.9980	1.43040
	2.00	2.8737	1.17254
	3.00	2.8226	1.05205
	4.00	2.5414	1.00853
	5.00	2.4661	1.21514

	Total	2.7386	1.19564
Valine (g)	1.00	4.3973	2.04517
	2.00	4.2885	1.73236
	3.00	4.1521	1.48327
	4.00	3.8195	1.48738
	5.00	3.6811	1.89559
	Total	4.0645	1.75534

animal fat (gram)	1.00	33.2604	22.55878
	2.00	29.2315	19.57020
	3.00	25.3307	12.37610
	4.00	23.2438	12.42606
	5.00	21.7335	14.20867
	Total	26.5457	17.14200
aMED Score	1.00	3.36	1.740
	2.00	4.59	1.970
	3.00	4.26	1.901
	4.00	4.41	1.962
	5.00	4.16	1.670
	Total	4.14	1.879
Ethanol Between 5-15 g/day Grade	1.00	.18	.390
	2.00	.28	.456
	3.00	.17	.377
	4.00	.15	.358
	5.00	.23	.424
	Total	.20	.401
Fat Intake Grade	1.00	.34	.479
	2.00	.54	.505
	3.00	.57	.501
	4.00	.56	.502
	5.00	.66	.479

	Total	.53	.500
Fish Grade	1.00	.41	.497
	2.00	.44	.502
	3.00	.50	.506
	4.00	.56	.502
	5.00	.43	.501
	Total	.47	.500
Fruit Grade	1.00	.41	.497
	2.00	.51	.506
	3.00	.50	.506
	4.00	.56	.502
	5.00	.50	.506
	Total	.50	.501
Legume Grade	1.00	.43	.501
	2.00	.54	.505
	3.00	.43	.501
	4.00	.61	.494
	5.00	.48	.505
	Total	.50	.501
Meat Grade	1.00	.36	.487
	2.00	.51	.506
	3.00	.33	.477
	4.00	.51	.506
	5.00	.48	.505
	Total	.44	.497
Nut/Seed Grade	1.00	.36	.487
	2.00	.67	.478
	3.00	.50	.506

	4.00	.41	.499
	5.00	.39	.493
	Total	.46	.500
Vegetable Grade	1.00	.39	.493
	2.00	.59	.498
	3.00	.67	.477
	4.00	.59	.499
	5.00	.48	.505
	Total	.54	.500
Whole Grain Grade	1.00	.48	.505
	2.00	.51	.506
	3.00	.60	.497
	4.00	.46	.505
	5.00	.52	.505
	Total	.51	.501
AOAC Determination of Total Fibre (g)	1.00	22.7213	10.52434
	2.00	27.6454	13.63418
	3.00	25.7279	12.19702
	4.00	26.6850	13.65110
	5.00	23.6293	20.14528
	Total	25.2071	14.46580
Aspartame (g)	1.00	.0349	.07953
	2.00	.0441	.11236
	3.00	.0260	.03691
	4.00	.0529	.08904
	5.00	.0463	.07672
	Total	.0408	.08191
Aspartic acid from Aspartame (g)	1.00	.0173	.04002
	2.00	.0215	.05637

	3.00	.0133	.01848
	4.00	.0260	.04456
	5.00	.0230	.03938
	Total	.0202	.04124
Phenylalanine from Aspartame (g)	1.00	.0173	.04002
	2.00	.0215	.05637
	3.00	.0133	.01848
	4.00	.0260	.04456
	5.00	.0230	.03938
	Total	.0202	.04124
Betaine, no supplements (mg)	1.00	105.2856	73.16336
	2.00	114.6495	71.48429
	3.00	106.4856	45.66042
	4.00	128.2193	107.93564
	5.00	106.6626	100.86181
	Total	112.0233	82.74567
Betane + Choline (mg)	1.00	460.5840	208.69478
	2.00	463.6805	189.26427
	3.00	463.2058	152.29288
	4.00	449.2588	178.62973
	5.00	436.4998	327.46581
	Total	454.3912	220.28512
Betane + Choline, no supplements (mg)	1.00	459.0324	207.69112
	2.00	458.7002	188.73742
	3.00	457.3577	146.78493
	4.00	448.1840	178.92250
	5.00	434.7476	327.63293
	Total	451.3902	219.32430

Caffeine (mg)	1.00	186.8364	139.52235
	2.00	186.3566	161.32581
	3.00	166.9614	124.00904
	4.00	137.6879	127.64796
	5.00	193.7652	160.95703
	Total	174.7635	143.79702
Calories (kcal)	1.00	1912.1125	701.63024
	2.00	2027.7805	810.05141
	3.00	1886.2335	618.98115
	4.00	1825.5671	676.62326
	5.00	1668.0720	478.50041
	Total	1860.3930	666.65743
Carbohydrate/kilocalorie	1.00	.4939	.08119
	2.00	.4852	.08405
	3.00	.4981	.08434
	4.00	.5082	.10015
	5.00	.5088	.07449
	Total	.4990	.08466
carbohydrates (gram)	1.00	235.2704	86.84527
	2.00	253.5176	122.57396
	3.00	235.0023	93.25178
	4.00	245.7783	120.56447
	5.00	235.0013	170.82647
	Total	240.6417	122.08135
Carbohydrates (g)	1.00	235.2704	86.84527
	2.00	253.5176	122.57396
	3.00	235.0023	93.25178
	4.00	245.7783	120.56447

	5.00	235.0013	170.82647
	Total	240.6417	122.08135
Estimated Free Carnitine (mg)	1.00	31.36224788000	22.105956280000
	2.00	26.45845418000	15.105571760000
	3.00	27.37522511000	18.124867670000
	4.00	24.64788202000	14.233599240000
	5.00	22.48237821000	18.131891820000
	Total	26.48269466000	17.946846490000
Total Fibre from Cereal (g)	1.00	6.1498	3.43341
	2.00	7.1976	4.65477
	3.00	6.0230	3.21994
	4.00	7.9962	6.59725
	5.00	5.9113	3.49780
	Total	6.6294	4.46043
cholesterol (mg)	1.00	228.2273	146.27633
	2.00	184.4690	97.03697
	3.00	194.1167	68.47843
	4.00	165.6286	84.59848
	5.00	170.8954	118.43345
	Total	188.9312	108.63016
Cholesterol (mg)	1.00	228.2273	146.27633
	2.00	184.4690	97.03697
	3.00	194.1167	68.47843

	4.00	165.6286	84.59848
	5.00	170.8954	118.43345
	Total	188.9312	108.63016
Total Choline, no supplements (mg)	1.00	353.7473	154.35387
	2.00	344.0515	132.44406
	3.00	350.8714	117.10763
	4.00	319.9655	109.58210
	5.00	328.0857	233.46392
	Total	339.3673	156.77006
Total Choline (mg)	1.00	355.2704	155.29048
	2.00	349.0059	134.86181
	3.00	356.6844	123.86724
	4.00	321.0098	109.59259
	5.00	329.8148	233.18412
	Total	342.3398	158.35968
Total Fibre from Cruciferous Veg (g)	1.00	1.0042	1.62355
	2.00	1.5768	1.44739
	3.00	1.1881	.92371
	4.00	1.1293	.79642
	5.00	1.7043	4.53871
	Total	1.3215	2.36181
Eggs	1.00	2.00	1.917
	2.00	1.55	2.375
	3.00	2.14	2.199
	4.00	1.29	2.110
	5.00	1.46	2.041
	Total	1.69	2.134
Eggs with Omegat 3 Fatty Acids	1.00	2.41	3.768
	2.00	2.18	3.605

	3.00	3.00	4.047
	4.00	2.19	3.658
	5.00	2.11	3.548
	Total	2.38	3.709
Eggstotal	1.00	4.4091	4.22791
	2.00	3.7436	5.03523
	3.00	5.1395	4.89694
	4.00	3.4762	4.62885
	5.00	3.5652	4.61231
	Total	4.0701	4.67736
Alcohol (g)	1.00	9.2471	15.32405
	2.00	15.3673	21.88613
	3.00	9.8109	17.29616
	4.00	14.0143	16.45076
	5.00	10.1798	14.06162
	Total	11.6356	17.12652
Ethanol Consuption	1.00	10.0845	18.38283
	2.00	13.9549	17.48756
	3.00	11.3117	16.82514
	4.00	10.4312	15.75601
	5.00	10.7709	14.56666
	Total	11.2602	16.54273
Animal Fat (g)	1.00	33.2604	22.55878
	2.00	29.2315	19.57020
	3.00	25.3307	12.37610
	4.00	23.2438	12.42606
	5.00	21.7335	14.20867
	Total	26.5457	17.14200
Fat/kilocalorie	1.00	.3269	.07869
	2.00	.3246	.06390
	3.00	.3177	.06784
	4.00	.2892	.07237
	5.00	.3018	.06422
	Total	.3121	.07049

Mono-Unsaturated Fats (g)	1.00	26.6347	14.73936
	2.00	28.9315	15.42242
	3.00	25.0437	11.93564
	4.00	22.5381	12.58298
	5.00	23.5098	16.81664
	Total	25.2981	14.49222
Poly-Unsaturated Fats (g)	1.00	16.4769	11.17235
	2.00	17.9083	9.57463
	3.00	15.8072	7.83748
	4.00	14.5733	7.79749
	5.00	15.2807	13.73627
	Total	15.9926	10.31743
Saturated Fats (g)	1.00	25.6420	16.48765
	2.00	23.7527	13.75022
	3.00	20.5463	8.16223
	4.00	18.7838	9.48215
	5.00	18.6000	13.16732
	Total	21.4551	12.82464
Total Fat (g)	1.00	75.0136	42.31450
	2.00	76.4771	38.99227
	3.00	66.9063	27.94849
	4.00	60.9662	30.59776
	5.00	62.2946	45.56044
	Total	68.2685	38.06492
Vegetable Fat (g)	1.00	41.7522	25.34033
	2.00	47.2451	25.51660
	3.00	41.5758	22.01167
	4.00	37.7219	21.36587
	5.00	40.5602	35.44003
	Total	41.7224	26.51601
Fish Consumption	1.00	4.66	7.820

	2.00	5.44	7.525
	3.00	5.17	5.495
	4.00	6.59	8.313
	5.00	4.64	5.288
	Total	5.28	6.949
Free Choline (mg)	1.00	83.6784	35.38211
	2.00	97.7017	45.84211
	3.00	88.4284	36.91255
	4.00	91.6126	33.39455
	5.00	91.3357	75.28539
	Total	90.4281	48.19116
Free Choline, no supplements (mg)	1.00	82.4127	34.26667
	2.00	92.9620	42.12063
	3.00	87.1360	36.59897
	4.00	90.8450	33.51353
	5.00	89.7983	75.29827
	Total	88.5395	47.28523
Total Fibre from Fruit (g)	1.00	5.3716	4.33715
	2.00	6.5624	4.46372
	3.00	6.8419	6.74908
	4.00	5.2340	4.00304
	5.00	5.3711	5.14338
	Total	5.8612	5.03924

Fruit Consumption	1.00	18.34	16.596
	2.00	18.21	15.142
	3.00	19.86	19.394
	4.00	20.78	19.748
	5.00	17.41	14.767
	Total	18.90	17.121
Fructose (g)	1.00	21.7313	11.90696

	2.00	25.0551	13.55792
	3.00	24.2707	16.94766
	4.00	22.8152	10.67046
	5.00	25.6674	36.47640
	Total	23.9067	20.48729
fat for frying: N/A	1.00	1.00	.000
	2.00	.	.
	3.00	1.00	.
	4.00	16.00	25.981
	5.00	1.00	.000
	Total	4.21	12.027
Follic Acid (B9, ug)	1.00	780.38	446.293
	2.00	878.39	515.276
	3.00	801.58	499.296
	4.00	945.21	497.072
	5.00	921.35	691.193
	Total	864.88	537.494
fat for frying: olive oil	1.00	1.00	.000
	2.00	1.00	.000
	3.00	1.00	.000
	4.00	1.00	.000
	5.00	1.00	.000
	Total	1.00	.000
fat for frying: veg oil	1.00	1.00	.000
	2.00	1.00	.000
	3.00	1.00	.000
	4.00	1.00	.000
	5.00	1.00	.000
	Total	1.00	.000
glucose (gm)	1.00	21.4122	10.95877
	2.00	23.0980	12.69168
	3.00	23.3323	16.98136
	4.00	23.1376	12.01889
	5.00	23.7491	29.57458
	Total	22.9406	17.90733

Choline from Glycerophosphocholine (mg)	1.00	69.1551	32.78085
	2.00	68.8478	33.93965
	3.00	70.0416	30.88359
	4.00	63.7669	35.83872
	5.00	62.3237	41.03453
	Total	66.7817	34.96241
Total Fibre from Legumes (g)	1.00	1.9798	1.34291
	2.00	2.7700	2.33633
	3.00	2.7421	2.17438
	4.00	2.7519	3.37253
	5.00	2.1389	1.54167
	Total	2.4633	2.25216
Legume Consumption	1.00	7.61	6.222
	2.00	10.03	8.190
	3.00	9.21	7.925
	4.00	13.83	11.885
	5.00	10.00	8.871
	Total	10.10	8.938
Meat Consumption	1.00	26.52	16.002
	2.00	26.08	19.943
	3.00	26.50	21.861
	4.00	28.34	24.912
	5.00	25.55	21.869
	Total	26.59	20.900
Mono-Unsaturated to Saturated Fat Ratio	1.00	1.0949	.23060
	2.00	1.2725	.40051
	3.00	1.2303	.34820
	4.00	1.2192	.27290
	5.00	1.3436	.48352

	Total	1.2313	.36491
monounsat fat (gram)	1.00	26.6347	14.73936
	2.00	28.9315	15.42242
	3.00	25.0437	11.93564
	4.00	22.5381	12.58298
	5.00	23.5098	16.81664
	Total	25.2981	14.49222
Nut Consumption	1.00	3.91	3.361
	2.00	6.74	5.533
	3.00	5.98	5.484
	4.00	4.61	4.811
	5.00	4.55	5.538
	Total	5.12	5.059
total Omega (EPA and DHA) (gram)	1.00	.2833	.30894
	2.00	.3515	.39070
	3.00	.3884	.35565
	4.00	.4440	.39008
	5.00	.3348	.29635
	Total	.3590	.34985
Omegat 3 Fatty Acid (g)	1.00	.2833	.30894
	2.00	.3515	.39070
	3.00	.3884	.35565
	4.00	.4440	.39008
	5.00	.3348	.29635
	Total	.3590	.34985
Choline from Phosphocholine (mg)	1.00	16.4573	7.07395
	2.00	17.6127	8.60674
	3.00	18.1233	7.27847

	4.00	16.2374	7.37861
	5.00	16.5730	17.19958
	Total	16.9877	10.33016
polyunsat fat (gram)	1.00	16.4769	11.17235
	2.00	17.9083	9.57463
	3.00	15.8072	7.83748
	4.00	14.5733	7.79749
	5.00	15.2807	13.73627
	Total	15.9926	10.31743
Crude AAs contributing to indole metabolites (g/day)	1.00	7.6251	3.55236
	2.00	7.4276	2.98150
	3.00	7.2419	2.61335
	4.00	6.6379	2.64314
	5.00	6.3800	3.23161
	Total	7.0568	3.04604
Crude TMA Precursors (mg/day)	1.00	496.9063	224.92297
	2.00	501.3069	191.00575
	3.00	481.1912	152.57152
	4.00	474.9596	187.11905
	5.00	462.8276	349.48996
	Total	483.1554	231.60677
protein (gram)	1.00	83.2031	38.75183
	2.00	82.3559	32.72237
	3.00	79.9133	27.61129
	4.00	74.1543	28.02093
	5.00	70.6252	37.31005
	Total	77.9735	33.40517
Protein (g)	1.00	83.2031	38.75183

	2.00	82.3559	32.72237
	3.00	79.9133	27.61129
	4.00	74.1543	28.02093
	5.00	70.6252	37.31005
	Total	77.9735	33.40517
Animal Protein (g)	1.00	56.3113	30.04778
	2.00	51.2251	23.89068
	3.00	50.9314	20.79364
	4.00	44.4805	17.88518
	5.00	44.0315	22.79271
	Total	49.3913	23.75985
Protein/kilocalorie	1.00	.1684	.03132
	2.00	.1630	.02872
	3.00	.1700	.02521
	4.00	.1617	.02838
	5.00	.1597	.03222
	Total	.1646	.02933
Choline from Phosphatidylcholine (mg)	1.00	166.4276	93.51389
	2.00	148.1651	65.11439
	3.00	163.1488	65.30780
	4.00	134.9476	56.05920
	5.00	144.9002	115.82879
	Total	151.6711	83.21959
Phosphatidylcholine, no supplements (mg)	1.00	166.1958	93.48570
	2.00	147.9722	65.20639
	3.00	158.6614	55.09316
	4.00	134.6995	56.00111
	5.00	144.7285	115.81070
	Total	150.6129	81.66243

Canola Oil	1.00	1.00	.000
	2.00	1.00	.000
	3.00	1.00	.000
	4.00	1.00	.000
	5.00	1.00	.000
	Total	1.00	.000
Sex	1.00	.33	.476
	2.00	.46	.504
	3.00	.40	.495
	4.00	.36	.483
	5.00	.50	.505
	Total	.41	.492
Choline from Sphingomyelin (mg)	1.00	19.5504	11.49657
	2.00	16.6778	8.04708
	3.00	16.9433	6.68395
	4.00	14.4433	6.18492
	5.00	14.6817	8.72247
	Total	16.4705	8.61528
Sucralose (g)	1.00	.5844	2.14674
	2.00	.7683	3.79955
	3.00	.3023	.90610
	4.00	.9552	2.36443
	5.00	.9680	2.37542
	Total	.7164	2.46402
Total Sugars (g)	1.00	105.7724	43.23796
	2.00	106.0356	51.27119
	3.00	108.4423	55.52132
	4.00	104.4610	51.92571
	5.00	111.4078	103.16065
	Total	107.2920	64.82392
total sugars (gm)	1.00	105.7724	43.23796
	2.00	106.0356	51.27119
	3.00	108.4423	55.52132
	4.00	104.4610	51.92571
	5.00	111.4078	103.16065

	Total	107.2920	64.82392
total fat (gram)	1.00	75.0136	42.31450
	2.00	76.4771	38.99227
	3.00	66.9063	27.94849
	4.00	60.9662	30.59776
	5.00	62.2946	45.56044
	Total	68.2685	38.06492
total trans fat; 2011	1.00	1.3569	.79746
	2.00	1.4005	.89353
	3.00	1.2044	.52229
	4.00	1.1850	.71889
	5.00	1.2043	1.04962
	Total	1.2693	.81611

Descriptives

		Mean	Std. Deviation
Vegetable oil	1.00	1.00	.000
	2.00	1.00	.000
	3.00	1.00	.000
	4.00	1.00	.000
	5.00	1.00	.000
	Total	1.00	.000
Total Fibre from Vegetable (g)	1.00	6.4589	4.27041
	2.00	8.8385	5.60332
	3.00	8.2147	4.47470
	4.00	8.7519	6.22246
	5.00	8.1863	12.02784
	Total	8.0664	7.18760
Vegetable Consumption	1.00	35.75	25.939
	2.00	49.85	36.087
	3.00	48.10	33.844
	4.00	53.49	41.139
	5.00	44.23	34.938
	Total	46.08	34.819
Vegetable Protein (g)	1.00	26.8911	11.61602
	2.00	31.1298	15.50529
	3.00	28.9828	12.36634

	4.00	29.6736	16.18149
	5.00	26.5933	18.50148
	Total	28.5818	15.02303
vegetable fat (gram)	1.00	41.7522	25.34033
	2.00	47.2451	25.51660
	3.00	41.5758	22.01167
	4.00	37.7219	21.36587
	5.00	40.5602	35.44003
	Total	41.7224	26.51601
Whole Grain Consumption	1.00	11.61	8.659
	2.00	13.74	11.042
	3.00	15.76	11.433
	4.00	14.59	15.406
	5.00	12.89	10.228
	Total	13.69	11.500

ANOVA

		Sig.
		.329
Alanine (g)	Between Groups	
		.298
Arginine (g)	Between Groups	
		.406
Aspartate (g)	Between Groups	.606
		.382
Cysteine (g)	Between Groups	
		.291
Glutamate (g)	Between Groups	
		.213
Glycine (g)	Between Groups	.245
		.237
Histidine (g)	Between Groups	
		.205
Isoleucine (g)	Between Groups	
		.180
Leucine (g)	Between Groups	.293
		.231
Lysine (g)	Between Groups	
		.272
Methionine (g)	Between Groups	
		.318
Phenylalanine (g)	Between Groups	.383
		.172
Proline (g)	Between Groups	
		.249
Serine (g)	Between Groups	

Threonine (g)	Between Groups	.009
Tryptophan (g)	Between Groups	.027
Tyrosine (g)	Between Groups	.570
Valine (g)	Between Groups	
animal fat (gram)	Between Groups	
aMED Score	Between Groups	
Ethanol Between 5-15 g/day Grade	Between Groups	
Fat Intake Grade	Between Groups	.045
Fish Grade	Between Groups	.632
Fruit Grade	Between Groups	.728
Legume Grade	Between Groups	.418
Meat Grade	Between Groups	.298
Nut/Seed Grade	Between Groups	.041
Vegetable Grade	Between Groups	.081
Whole Grain Grade	Between Groups	.781
AOAC Determination of Total Fibre (g)	Between Groups	.476
Aspartame (g)	Between Groups	.593
Aspartic acid from Aspartame (g)	Between Groups	.646
Phenylalanine from Aspartame (g)	Between Groups	.646
Betaine, no supplements (mg)	Between Groups	.678
Betane + Choline (mg)	Between Groups	.974
Betane + Choline, no supplements (mg)	Between Groups	.982
Caffeine (mg)	Between Groups	.368
Calories (kcal)	Between Groups	.151

Carbohydrate/kilocalorie	Between Groups	.684
carbohydrates (gram)	Between Groups	.939
Carbohydrates (g)	Between Groups	.939
Estimated Free Carnitine (mg)	Between Groups	.201
Total Fibre from Cereal (g)	Between Groups	.129
cholesterol (mg)	Between Groups	.053
Cholesterol (mg)	Between Groups	.053
Total Choline, no supplements (mg)	Between Groups	.824
Total Choline (mg)	Between Groups	.780
Total Fibre from Cruciferous Veg (g)	Between Groups	.576
Eggs	Between Groups	.281
Eggs with Omegat 3 Fatty Acids	Between Groups	.799
Eggstotal	Between Groups	.425
Alcohol (g)	Between Groups	.350
Ethanol Consuption	Between Groups	.847
Animal Fat (g)	Between Groups	.009
Fat/kilocalorie	Between Groups	.065
Mono-Unsaturated Fats (g)	Between Groups	.269
Poly-Unsaturated Fats (g)	Between Groups	.641
Saturated Fats (g)	Between Groups	.032
Total Fat (g)	Between Groups	.195
Vegetable Fat (g)	Between Groups	.591
Fish Consumption	Between Groups	.702
Free Choline (mg)	Between Groups	.752
Free Choline, no supplements (mg)	Between Groups	.866

Total Fibre from Fruit (g)	Between Groups	.418
Fruit Consumption	Between Groups	.901
Fructose (g)	Between Groups	.895
fat for frying: N/A	Between Groups	.325
Follic Acid (B9, ug)	Between Groups	.531
fat for frying: olive oil	Between Groups	.
fat for frying: veg oil	Between Groups	.
glucose (gm)	Between Groups	.979
Choline from Glycerophosphocholine (mg)	Between Groups	.779
Total Fibre from Legumes (g)	Between Groups	.274
Legume Consumption	Between Groups	.025
Meat Consumption	Between Groups	.981
Mono-Unsaturated to Saturated Fat Ratio	Between Groups	.027
monounsat fat (gram)	Between Groups	.269
Nut Consumption	Between Groups	.067
total Omega (EPA and DHA) (gram)	Between Groups	.275
Omegat 3 Fatty Acid (g)	Between Groups	.275
Choline from Phosphocholine (mg)	Between Groups	.899
polyunsat fat (gram)	Between Groups	.641
Crude AAs contributing to indole metabolites (g/day)	Between Groups	.250
Crude TMA Precursors (mg/day)	Between Groups	.941
protein (gram)	Between Groups	.318
Protein (g)	Between Groups	.318
Animal Protein (g)	Between Groups	.077

Protein/kilocalorie	Between Groups	.410
Choline from Phosphatidylcholine (mg)	Between Groups	.368
Phosphatidylcholine, no supplements (mg)	Between Groups	.418
Canola Oil	Between Groups	.
Sex	Between Groups	.385
Choline from Sphingomyelin (mg)	Between Groups	.035
Sucralose (g)	Between Groups	.696
Total Sugars (g)	Between Groups	.989
total sugars (gm)	Between Groups	.989
total fat (gram)	Between Groups	.195
total trans fat; 2011	Between Groups	.632
Total Fibre from Vegetable (g)		.539
Vegetable Consumption		.167
Vegetable Protein (g)		.594
vegetable fat (gram)		.591
Whole Grain Consumption		.518
